# Supplementary material for: Heparin-based hydrogel scaffolding alters the transcriptomic profile and increases the chemoresistance of MDA-MB-231 triple-negative breast cancer cells
Source: Biomater Sci. 2020 Feb 13;8(10):2786–96. doi: 10.1039/c9bm01481k (PMC7497406; doi:10.1039/c9bm01481k)
Supplement: Supplementary file 2 [file BM-008-C9BM01481K-s002.zip › Supplementary File 4/HP-B hydrogel + EGF (s)vControl/Pathways/my_analysis.Gsea.1545201170987/HALLMARK_ESTROGEN_RESPONSE_EARLY.html]

Details for gene set HALLMARK\_ESTROGEN\_RESPONSE\_EARLY[GSEA]

|  || Dataset | expr.class.cls#HYDEGF\_versus\_CONTROL.class.cls#HYDEGF\_versus\_CONTROL\_repos |
| Phenotype | class.cls#HYDEGF\_versus\_CONTROL\_repos |
| Upregulated in class | CONTROL |
| GeneSet | HALLMARK\_ESTROGEN\_RESPONSE\_EARLY |
| Enrichment Score (ES) | -0.26537955 |
| Normalized Enrichment Score (NES) | -1.2612407 |
| Nominal p-value | 0.04368932 |
| FDR q-value | 0.103462085 |
| FWER p-Value | 0.801 |
Table: GSEA Results Summary

  

Fig 1: Enrichment plot: HALLMARK\_ESTROGEN\_RESPONSE\_EARLY      
 Profile of the Running ES Score & Positions of GeneSet Members on the Rank Ordered List

  

| PROBE | DESCRIPTION (from dataset) | GENE SYMBOL | GENE\_TITLE | RANK IN GENE LIST | RANK METRIC SCORE | RUNNING ES | CORE ENRICHMENT || 1 | RASGRP1 | na |  |  | 87 | 2.987 | 0.0169 | No |
| 2 | SLC7A5 | na |  |  | 239 | 2.501 | 0.0269 | No |
| 3 | RRP12 | na |  |  | 389 | 2.244 | 0.0352 | No |
| 4 | SLC22A5 | na |  |  | 456 | 2.172 | 0.0473 | No |
| 5 | SH3BP5 | na |  |  | 626 | 1.980 | 0.0526 | No |
| 6 | DLC1 | na |  |  | 642 | 1.960 | 0.0659 | No |
| 7 | MYBBP1A | na |  |  | 667 | 1.933 | 0.0785 | No |
| 8 | CYP26B1 | na |  |  | 792 | 1.826 | 0.0851 | No |
| 9 | WWC1 | na |  |  | 850 | 1.783 | 0.0949 | No |
| 10 | SCARB1 | na |  |  | 994 | 1.690 | 0.0995 | No |
| 11 | SYNGR1 | na |  |  | 1499 | 1.433 | 0.0833 | No |
| 12 | RAPGEFL1 | na |  |  | 1545 | 1.411 | 0.0911 | No |
| 13 | AKAP1 | na |  |  | 1739 | 1.345 | 0.0906 | No |
| 14 | ITPK1 | na |  |  | 1781 | 1.335 | 0.0981 | No |
| 15 | LRIG1 | na |  |  | 1869 | 1.304 | 0.1028 | No |
| 16 | FOS | na |  |  | 2215 | 1.207 | 0.0934 | No |
| 17 | PODXL | na |  |  | 2293 | 1.182 | 0.0978 | No |
| 18 | ASB13 | na |  |  | 2621 | 1.099 | 0.0885 | No |
| 19 | FARP1 | na |  |  | 2623 | 1.098 | 0.0963 | No |
| 20 | CCND1 | na |  |  | 2684 | 1.084 | 0.1010 | No |
| 21 | STC2 | na |  |  | 3009 | 1.002 | 0.0911 | No |
| 22 | SLC1A4 | na |  |  | 3358 | 0.931 | 0.0795 | No |
| 23 | TTC39A | na |  |  | 3369 | 0.928 | 0.0857 | No |
| 24 | THSD4 | na |  |  | 3399 | 0.922 | 0.0908 | No |
| 25 | FASN | na |  |  | 3578 | 0.888 | 0.0878 | No |
| 26 | FHL2 | na |  |  | 3633 | 0.879 | 0.0913 | No |
| 27 | MED13L | na |  |  | 3701 | 0.865 | 0.0939 | No |
| 28 | NAV2 | na |  |  | 3798 | 0.848 | 0.0950 | No |
| 29 | NCOR2 | na |  |  | 3891 | 0.831 | 0.0961 | No |
| 30 | UNC119 | na |  |  | 4234 | 0.777 | 0.0837 | No |
| 31 | MYC | na |  |  | 4314 | 0.764 | 0.0851 | No |
| 32 | SIAH2 | na |  |  | 4404 | 0.748 | 0.0858 | No |
| 33 | MLPH | na |  |  | 4410 | 0.748 | 0.0909 | No |
| 34 | MREG | na |  |  | 4621 | 0.717 | 0.0850 | No |
| 35 | CHPT1 | na |  |  | 4713 | 0.704 | 0.0852 | No |
| 36 | TIPARP | na |  |  | 4770 | 0.692 | 0.0873 | No |
| 37 | MED24 | na |  |  | 4824 | 0.683 | 0.0894 | No |
| 38 | JAK2 | na |  |  | 4877 | 0.671 | 0.0915 | No |
| 39 | AMFR | na |  |  | 4909 | 0.667 | 0.0946 | No |
| 40 | ADCY9 | na |  |  | 4979 | 0.655 | 0.0957 | No |
| 41 | NADSYN1 | na |  |  | 5166 | 0.626 | 0.0904 | No |
| 42 | KDM4B | na |  |  | 5281 | 0.609 | 0.0888 | No |
| 43 | CD44 | na |  |  | 5293 | 0.607 | 0.0926 | No |
| 44 | TBC1D30 | na |  |  | 5358 | 0.596 | 0.0935 | No |
| 45 | BCL2 | na |  |  | 5367 | 0.594 | 0.0973 | No |
| 46 | SYT12 | na |  |  | 5533 | 0.572 | 0.0928 | No |
| 47 | RBBP8 | na |  |  | 5572 | 0.567 | 0.0948 | No |
| 48 | HES1 | na |  |  | 5622 | 0.558 | 0.0963 | No |
| 49 | FRK | na |  |  | 5640 | 0.556 | 0.0994 | No |
| 50 | KAZN | na |  |  | 5727 | 0.542 | 0.0988 | No |
| 51 | ISG20L2 | na |  |  | 5827 | 0.528 | 0.0973 | No |
| 52 | TFAP2C | na |  |  | 6119 | 0.487 | 0.0855 | No |
| 53 | CELSR2 | na |  |  | 6140 | 0.484 | 0.0880 | No |
| 54 | MYBL1 | na |  |  | 6260 | 0.468 | 0.0851 | No |
| 55 | SLC1A1 | na |  |  | 6381 | 0.452 | 0.0820 | No |
| 56 | MYOF | na |  |  | 6482 | 0.438 | 0.0799 | No |
| 57 | PPIF | na |  |  | 6528 | 0.434 | 0.0806 | No |
| 58 | BAG1 | na |  |  | 6712 | 0.409 | 0.0740 | No |
| 59 | ESRP2 | na |  |  | 6731 | 0.406 | 0.0759 | No |
| 60 | SEMA3B | na |  |  | 6755 | 0.403 | 0.0776 | No |
| 61 | RHOBTB3 | na |  |  | 6764 | 0.400 | 0.0801 | No |
| 62 | CELSR1 | na |  |  | 6956 | 0.378 | 0.0727 | No |
| 63 | IGF1R | na |  |  | 6976 | 0.375 | 0.0744 | No |
| 64 | NXT1 | na |  |  | 7070 | 0.363 | 0.0722 | No |
| 65 | DHRS2 | na |  |  | 7251 | 0.337 | 0.0651 | No |
| 66 | ABLIM1 | na |  |  | 7413 | 0.315 | 0.0589 | No |
| 67 | SYBU | na |  |  | 7460 | 0.310 | 0.0587 | No |
| 68 | PDZK1 | na |  |  | 7616 | 0.287 | 0.0526 | No |
| 69 | MICB | na |  |  | 7714 | 0.277 | 0.0495 | No |
| 70 | KRT15 | na |  |  | 7721 | 0.276 | 0.0512 | No |
| 71 | EGR3 | na |  |  | 7749 | 0.271 | 0.0517 | No |
| 72 | FKBP5 | na |  |  | 7831 | 0.259 | 0.0493 | No |
| 73 | ADCY1 | na |  |  | 7850 | 0.257 | 0.0502 | No |
| 74 | KLF4 | na |  |  | 7914 | 0.254 | 0.0487 | No |
| 75 | WFS1 | na |  |  | 7951 | 0.249 | 0.0486 | No |
| 76 | CLIC3 | na |  |  | 8093 | 0.230 | 0.0429 | No |
| 77 | MYB | na |  |  | 8130 | 0.230 | 0.0426 | No |
| 78 | KRT8 | na |  |  | 8186 | 0.226 | 0.0414 | No |
| 79 | SNX24 | na |  |  | 8213 | 0.221 | 0.0416 | No |
| 80 | HR | na |  |  | 8338 | 0.203 | 0.0365 | No |
| 81 | SLC37A1 | na |  |  | 8364 | 0.200 | 0.0366 | No |
| 82 | MAPT | na |  |  | 8425 | 0.198 | 0.0349 | No |
| 83 | ALDH3B1 | na |  |  | 8580 | 0.177 | 0.0281 | No |
| 84 | INHBB | na |  |  | 8618 | 0.169 | 0.0274 | No |
| 85 | ZNF185 | na |  |  | 8751 | 0.158 | 0.0216 | No |
| 86 | ABCA3 | na |  |  | 8759 | 0.156 | 0.0223 | No |
| 87 | RARA | na |  |  | 8781 | 0.154 | 0.0223 | No |
| 88 | SFN | na |  |  | 9071 | 0.119 | 0.0080 | No |
| 89 | AFF1 | na |  |  | 9279 | 0.091 | -0.0023 | No |
| 90 | INPP5F | na |  |  | 9345 | 0.082 | -0.0051 | No |
| 91 | FAM102A | na |  |  | 9501 | 0.075 | -0.0127 | No |
| 92 | TPBG | na |  |  | 9534 | 0.070 | -0.0139 | No |
| 93 | TUBB2B | na |  |  | 10009 | 0.019 | -0.0386 | No |
| 94 | SLC19A2 | na |  |  | 10072 | 0.009 | -0.0418 | No |
| 95 | SLC26A2 | na |  |  | 10074 | 0.009 | -0.0418 | No |
| 96 | REEP1 | na |  |  | 10198 | -0.007 | -0.0482 | No |
| 97 | RHOD | na |  |  | 10325 | -0.026 | -0.0547 | No |
| 98 | TPD52L1 | na |  |  | 10368 | -0.032 | -0.0566 | No |
| 99 | CANT1 | na |  |  | 10869 | -0.089 | -0.0823 | No |
| 100 | SLC7A2 | na |  |  | 10928 | -0.099 | -0.0846 | No |
| 101 | OLFML3 | na |  |  | 11010 | -0.108 | -0.0881 | No |
| 102 | TJP3 | na |  |  | 11329 | -0.139 | -0.1038 | No |
| 103 | KRT18 | na |  |  | 11421 | -0.149 | -0.1075 | No |
| 104 | FOXC1 | na |  |  | 11477 | -0.160 | -0.1093 | No |
| 105 | GAB2 | na |  |  | 11547 | -0.167 | -0.1117 | No |
| 106 | FKBP4 | na |  |  | 11609 | -0.176 | -0.1136 | No |
| 107 | PAPSS2 | na |  |  | 11849 | -0.211 | -0.1247 | No |
| 108 | SVIL | na |  |  | 12087 | -0.231 | -0.1355 | No |
| 109 | DEPTOR | na |  |  | 12115 | -0.234 | -0.1352 | No |
| 110 | P2RY2 | na |  |  | 12210 | -0.245 | -0.1384 | No |
| 111 | ADD3 | na |  |  | 12405 | -0.273 | -0.1466 | No |
| 112 | RPS6KA2 | na |  |  | 12594 | -0.297 | -0.1544 | No |
| 113 | PEX11A | na |  |  | 12605 | -0.299 | -0.1528 | No |
| 114 | RAB17 | na |  |  | 12738 | -0.320 | -0.1574 | No |
| 115 | AR | na |  |  | 12810 | -0.330 | -0.1588 | No |
| 116 | SLC2A1 | na |  |  | 13117 | -0.356 | -0.1723 | No |
| 117 | MAST4 | na |  |  | 13259 | -0.379 | -0.1770 | No |
| 118 | IL17RB | na |  |  | 13275 | -0.382 | -0.1750 | No |
| 119 | ABHD2 | na |  |  | 13290 | -0.384 | -0.1730 | No |
| 120 | PDLIM3 | na |  |  | 13293 | -0.385 | -0.1703 | No |
| 121 | PMAIP1 | na |  |  | 13499 | -0.413 | -0.1782 | No |
| 122 | GLA | na |  |  | 13717 | -0.444 | -0.1864 | No |
| 123 | TGIF2 | na |  |  | 13824 | -0.462 | -0.1886 | No |
| 124 | BCL11B | na |  |  | 14657 | -0.582 | -0.2282 | No |
| 125 | TMPRSS3 | na |  |  | 15104 | -0.654 | -0.2469 | No |
| 126 | AQP3 | na |  |  | 15228 | -0.674 | -0.2485 | No |
| 127 | SLC9A3R1 | na |  |  | 15259 | -0.680 | -0.2452 | No |
| 128 | OPN3 | na |  |  | 15285 | -0.684 | -0.2416 | No |
| 129 | ENDOD1 | na |  |  | 15520 | -0.725 | -0.2487 | No |
| 130 | NBL1 | na |  |  | 15591 | -0.741 | -0.2471 | No |
| 131 | ARL3 | na |  |  | 15839 | -0.790 | -0.2544 | No |
| 132 | XBP1 | na |  |  | 16049 | -0.833 | -0.2594 | Yes |
| 133 | FDFT1 | na |  |  | 16053 | -0.834 | -0.2536 | Yes |
| 134 | TSKU | na |  |  | 16131 | -0.853 | -0.2515 | Yes |
| 135 | TMEM164 | na |  |  | 16170 | -0.863 | -0.2473 | Yes |
| 136 | TIAM1 | na |  |  | 16222 | -0.879 | -0.2437 | Yes |
| 137 | HSPB8 | na |  |  | 16267 | -0.891 | -0.2396 | Yes |
| 138 | SLC39A6 | na |  |  | 16293 | -0.896 | -0.2345 | Yes |
| 139 | DHCR7 | na |  |  | 16459 | -0.938 | -0.2364 | Yes |
| 140 | GJA1 | na |  |  | 16597 | -0.968 | -0.2367 | Yes |
| 141 | PRSS23 | na |  |  | 16650 | -0.985 | -0.2323 | Yes |
| 142 | BHLHE40 | na |  |  | 16798 | -1.027 | -0.2327 | Yes |
| 143 | TGM2 | na |  |  | 16812 | -1.030 | -0.2260 | Yes |
| 144 | ELF3 | na |  |  | 16854 | -1.043 | -0.2206 | Yes |
| 145 | KLF10 | na |  |  | 16932 | -1.064 | -0.2170 | Yes |
| 146 | KRT19 | na |  |  | 17022 | -1.096 | -0.2138 | Yes |
| 147 | ELOVL5 | na |  |  | 17447 | -1.235 | -0.2273 | Yes |
| 148 | SCNN1A | na |  |  | 17480 | -1.250 | -0.2200 | Yes |
| 149 | ELF1 | na |  |  | 17482 | -1.252 | -0.2110 | Yes |
| 150 | BLVRB | na |  |  | 17508 | -1.262 | -0.2033 | Yes |
| 151 | PTGES | na |  |  | 17682 | -1.324 | -0.2029 | Yes |
| 152 | UGCG | na |  |  | 17687 | -1.326 | -0.1936 | Yes |
| 153 | B4GALT1 | na |  |  | 18015 | -1.493 | -0.2000 | Yes |
| 154 | CLDN7 | na |  |  | 18127 | -1.564 | -0.1946 | Yes |
| 155 | SEC14L2 | na |  |  | 18208 | -1.605 | -0.1873 | Yes |
| 156 | CALB2 | na |  |  | 18210 | -1.607 | -0.1758 | Yes |
| 157 | DHRS3 | na |  |  | 18222 | -1.612 | -0.1648 | Yes |
| 158 | TOB1 | na |  |  | 18307 | -1.689 | -0.1571 | Yes |
| 159 | TFF1 | na |  |  | 18353 | -1.713 | -0.1472 | Yes |
| 160 | PLA2G16 | na |  |  | 18422 | -1.773 | -0.1380 | Yes |
| 161 | IL6ST | na |  |  | 18442 | -1.792 | -0.1262 | Yes |
| 162 | ANXA9 | na |  |  | 18600 | -1.926 | -0.1206 | Yes |
| 163 | CISH | na |  |  | 18778 | -2.156 | -0.1144 | Yes |
| 164 | ABAT | na |  |  | 18799 | -2.192 | -0.0997 | Yes |
| 165 | NRIP1 | na |  |  | 18832 | -2.251 | -0.0853 | Yes |
| 166 | AREG | na |  |  | 18856 | -2.288 | -0.0700 | Yes |
| 167 | RAB31 | na |  |  | 19005 | -2.597 | -0.0592 | Yes |
| 168 | WISP2 | na |  |  | 19077 | -2.817 | -0.0427 | Yes |
| 169 | CA12 | na |  |  | 19153 | -3.311 | -0.0228 | Yes |
| 170 | DYNLT3 | na |  |  | 19169 | -3.505 | 0.0015 | Yes |
Table: GSEA details [plain text format]

  

Fig 2: HALLMARK\_ESTROGEN\_RESPONSE\_EARLY      
 Blue-Pink O' Gram in the Space of the Analyzed GeneSet

  

Fig 3: HALLMARK\_ESTROGEN\_RESPONSE\_EARLY: Random ES distribution      
 Gene set null distribution of ES for **HALLMARK\_ESTROGEN\_RESPONSE\_EARLY**
